# Occurrence of Aflatoxin M_1_ in Milk Consumed in Tirana, Albania, and Health Risk Assessment in Different Population Groups

**DOI:** 10.3390/toxins17070315

**Published:** 2025-06-21

**Authors:** Andrin Tahiri, Josif Risto, Lorena Mato, Alma Cani, Dritan Topi

**Affiliations:** 1 Medical Service of Toxicology and Addictology, ‘Mother Teresa’ University Hospital Center, 1000 Tirana, Albania; andrin.tahiri@qsut.gov.al; 2 Department of Pharmacy, Faculty of Medical Sciences, Luarasi University, Rruga e Elbasanit Street, 1003 Tirana, Albania; josif.risto@uniluarasi.edu.al; 3 Department of Chemistry, Faculty of Natural Sciences, University of Tirana, Blvd. Zogu 1, No. 25, 1001 Tirana, Albania; l.mato@umb.edu.al; 4 Department of Surgery, Faculty of Medicine, University of Medicine, Tirana (UMT), 1000 Tirana, Albania; alma.cani@qsut.gov.al

**Keywords:** milk, aflatoxin M1, HPLC-FLD, exposure assessment, risk assessment, Albania

## Abstract

This study evaluated the prevalence of aflatoxin M1 (AFM1) in milk marketed in Tirana, Albania, along with dietary exposure and associated potential health risks. The World Health Organization has categorized Albania in cluster G02 of GEMS/FOOD, highlighting that milk is a staple in the Albanian diet, which points to a possible health risk. A total of 141 milk samples, comprising both Ultra-High Temperature (UHT) and pasteurized types, were collected from local markets in Tirana and analyzed from March 2023 to February 2024. The determination of AFM1 levels was carried out using High-Pressure Liquid Chromatography with a Fluorescence Detector (HPLC-FLD), a precise and dependable technique for identifying and measuring aflatoxins in food products. Aflatoxin M1 was found in 62.4% of the milk samples, with 26.2% surpassing the European Union’s maximum residue levels (MRL). The mean AFM1 concentrations were 58.8 ± 95.8 ng/kg, reaching a maximum level of 399.0 ng/kg. The Estimated Daily Intake (EDI) for various groups—toddlers, children, adolescents, and adults—was determined to be 2.161, 1.297, 0.519, and 0.370 ng/kg of body weight per day, respectively. The Hazard Index (HI), derived from the AFM1 exposure for four population groups, was 10.81 (toddlers), 6.48 (children), 2.59 (adolescents), and 1.85 (adults). The Margin of Exposure (MoE) was 1.85, 3.08, 7.71, and 10.81, respectively. The incidence of hepatocellular carcinoma (HCC) per 100,000 people in the four groups was 0.034, 0.021, 0.008, and 0.006, respectively. The study is the first comprehensive evaluation of AFM1 prevalence, highlighting the potential risks associated with milk consumption, as milk is a dietary staple in Albanian households. It addresses a critical public health concern regarding aflatoxin M1 (AFM1) contamination in milk consumed in Tirana, Albania, by highlighting the need for ongoing monitoring, regulatory measures, and educational outreach to enhance food safety and safeguard public health in Albania, as well as in other regions facing similar concerns.

## 1. Introduction

Milk is a food that has significantly influenced our metabolism and provides nourishment to humans from birth to old age. Dairy products are a crucial component of diets worldwide, spanning all continents [1]. As a product that undergoes various production stages—from farms to processing facilities and ultimately to consumers—milk is a significant pathway for human exposure to food contaminants, including mycotoxins [2]. Mycotoxins are secondary metabolites produced by molds, and their worldwide occurrence is well-documented, making them a focus for food regulatory bodies [3]. Eating foods contaminated with mycotoxins raises significant health concerns. Among these, aflatoxins are prominent as natural food contaminants, particularly aflatoxin B1 (AFB1). AFB1 is the most toxic of over twenty known aflatoxins, produced by the fungi *Aspergillus flavus* and *A. parasiticus*, commonly found in poorly dried cereal grains, maize, groundnuts, rice, and cottonseed [4,5]. AFB1 is a major contaminant in food and feed, affecting various animal feeds and leading to significant economic repercussions [6]. Lactating animals metabolize AFB1 in the liver via microsomal cytochrome P450, converting it to aflatoxin M1 (AFM1) [4,7]. AFM1 is recognized as a significant contaminant in milk and dairy products [8]. Exposure to AFM1 can cause both acute and chronic toxicosis [9]. After consuming AFB1-contaminated food, metabolic processes generate reactive metabolites, such as AFB1-8,9-epoxide, which can exert substantial biochemical effects and interact with cellular organelles and macromolecules. Acute aflatoxicosis may result from the protein-binding properties of bioactive AFB1 metabolites in the liver, potentially leading to organ failure. Additionally, oxidative damage to cells, tissues, and DNA may result in AFB1-mediated cytotoxicity and carcinogenic effects [10].

Like AFB1, AFM1 causes adverse effects on human health, such as carcinogenicity, mutagenicity, teratogenicity, hepatotoxicity, neurotoxicity, genotoxicity, and immunosuppression [2,4]. Initially classified as a 2B category, probable human carcinogen, it has recently been reclassified as a category 1 human carcinogen [10], with a carcinogenic scale of 10% of AFB1 [11].

The presence of AFM1 in milk and dairy products, even at very low levels, represents a chronic concern for public health, particularly for infants, children, and adolescents [4,12,13]. AFM1 contamination in milk and dairy products varies based on geography, environmental and climate conditions, and the country’s level of development [14].

Beyond direct human exposure to AFB1-contaminated foods, there is significant concern regarding AFB1-contaminated feeds, which indicates the metabolite transfer to milk and dairy products worldwide. Conventional milk treatments, such as pasteurization and ultra-high-temperature methods, are ineffective in removing AFM1 from milk [15]. Aflatoxins collectively heighten the risk of hepatocellular carcinoma (HCC), the fifth most prevalent cancer globally. Liu and Wu’s study (2015) [16] estimated that approximately 5–28% of HCC cases worldwide are associated with aflatoxin exposure, primarily through dietary intake. Besides setting regulatory limits for mycotoxins, performing health risk assessments concerning dietary exposure in the population is vital. The low-dose extrapolation method, introduced in 1997 by the Joint Expert Committee on Food Additives (JECFA), and the Margin of Exposure (MoE) approach, proposed during the 64th JECFA meeting in 2005, are recommended and extensively used worldwide to assess the risk of dietary mycotoxin exposure [4,17]. Risk characterization of potential adverse effects from AFM1 exposure in milk indicates a significant impact on consumer health. EFSA has suggested the use of the Hazard Index (HI) and Margin of Exposure (MoE) [18]. Research on AFM1 levels in Albanian milk and dairy products is scarce. This study investigates the presence of AFM1 in consumed milk in relation to dietary intake and associated cancer risks among consumers from various groups in Tirana, Albania. Addressing AFM1 contamination in milk and other dairy products is crucial for managing mycotoxin risks.

## 2. Results and Discussion

### 2.1. AFM1 Occurrence in Analyzed Milk

Milk production is a vital sector of the agricultural industry in Albania. According to FAOSTAT, the country’s milk production quantity was 788,307 tons in 2023, making it the top-producing commodity [19]. The milk commodity showcases the country’s significant potential, positioning it as the third-largest producer among Balkan countries [19]. In terms of economic revenue, milk production reached USD 328,055 million, accounting for 27.8% of the agricultural sector’s total revenue. Between 2018 and 2023, the country’s milk industry saw a 20% decline in milk production.

This study examined the occurrence of AFM1 in milk marketed and consumed by different age groups residing in Tirana. Of the 141 samples analyzed, 62.4% were found to be contaminated. Among these, 26.2% exceeded the EU maximum residue limit (MRL) of 0.05 µg/kg (EU Regulation, 915/2023 [20]. Milk samples in the 10–50 ng/kg range accounted for 21.3%, while those in the 3–10 ng/kg range comprised 14.9%, with a limit of detection (LOD) of 3 ng/kg. A prior study from 2019–2020 reported a 52.10% AFM1 contamination rate, with mean and maximum values of 22 ng/kg and 217 ng/kg, respectively (Table 1). Additionally, 5.9% exceeded the EU maximum residue limit (MRL).

In Albania, studies analyzing samples from 2014 to 2015 reported aflatoxin contamination in corn and wheat [21], as well as findings from the 2022 harvest year [22]. This situation raises concerns about feed safety, particularly regarding AFM1 contamination in milk from lactating animals that consume maize contaminated with aflatoxins.

### 2.2. Worldwide AFM1 Occurrence in Milk

The influence of climate change on aflatoxin contamination in maize in Southern Europe first became evident in the 2000s [4]. Evidence of AFM1 contamination in milk and dairy products is reported from various European Union countries, including Italy [14,18,23], Croatia [24], Spain [25], and Hungary [26], as well as from the Balkan region, covering Bosnia and Herzegovina [24], Kosovo [27,28], North Macedonia [29], Serbia [30,31], Turkey [32], and Albania [33].

Several publications report a high incidence of AFM1 in raw milk worldwide, threatening public health [15,34,35,36,37]. These data suggest a correlation with aflatoxin-contaminated feed, indicating that developing countries have higher incidence rates than developed economies. Reports from the African continent, including Ethiopia [38], Kenya [39,40], Ghana [41], and Nigeria [42], present a high alert scale. AFM1 incidence can be as high as 100% in milk samples from several countries. Maximum levels were observed in Ethiopia (4980 ng/kg), Kenya (4563 ng/kg), Ghana (3520 ng/kg), Egypt (8000 ng/kg), Sudan (6900 ng/kg), and Nigeria (2040 ng/kg) [13].

Reports on AFM1 contamination across Asia, including China [43], India [44], Iran [1,8,45], and Pakistan [46], highlight climatic conditions and lactating animals’ exposure to contaminated feed as primary factors. A review found the average AFM1 contamination in Iran was 77 ± 159 ng/kg, exceeding EU MRL [45]. Studies found a 64% incidence with a mean concentration of 39.7 ng/kg in Iranian raw milk, with 25% of samples exceeding the EU MRL (0.05 µg/kg) [8]. Seasonal variation affects AFM1 concentrations due to higher compound feed use in colder periods [20]. Overall, the AFM1 contamination incidence in milk samples was 95%, ranging from 2.02 to 189.3 ng/kg. In Pakistan, AFM1 detection in milk and dairy products has increased, with 71% of samples contaminated and 58% exceeding the EU MRL [34]. Ahmad et al. (2019) [46] reported a 91.4% incidence in Lahore’s urban milk, peaking at 554 ng/kg, with 72.0% exceeding the EU MRL. In India, a study indicated 100% incidence, maximum at 1116 ng/kg, with 70% over EU MRL [44]. In China, winter unpasteurized milk AFM1 was higher (123 ng/kg), with a 75.0% incidence [47].

Reports from various countries in South America, including Argentina [48] and Brazil [49], indicate that AFM1 contamination of milk and dairy products presents a significant global risk to human health. In Brazil, elevated AFM1 levels were observed in milk during the dry season. Consequently, these authors recommend that compulsory monitoring for AFM1 in milk in tropical countries, particularly during dry periods, should be supplemented by the adoption of good agricultural practices to prevent and reduce AFM1 contamination in animal feed.

The presence of AFM1 in Southern European milk indicates a persistent contamination issue. In the Marche region of Central Italy, AFM1 contamination reached 100%, but no samples exceeded the EU maximum residue limit (MRL) [23]. From 2013 to 2018, only 0.2% of samples exceeded the EU MRL across all Italian regions producing milk. AFM1 and its incidence in milk consumed in various countries in the region remain a persistent issue. A study on AFM1 in milk and dairy products in North Macedonia revealed a 66.8% incidence, with 24.2% exceeding the EU MRL [29]. In Croatia, average AFM1 concentrations varied significantly in cow’s milk [24]. Turkey reported an AFM1 incidence of 89.2% and maximum levels of 78.69 ng/kg [32]. Both studies demonstrated that 3.3% and 3.4% of samples exceeded the EU MRL, indicating effective monitoring. Serbia reported alarming AFM1 findings in 2013, with a 95% incidence and levels reaching up to 900 ng/kg, with 75% exceeding the EU MRL [30]. Data from this decade reveal an 80.8% incidence and peak levels of 1260 ng/kg, with 25.6% exceeding the EU MRL, suggesting climate change challenges in the Balkans [31,50]. A recent study from Kosovo found that the average AFM1 level in yogurt was 71 ng/kg, with 89.0% exceeding the EU MRL [27]. In Hungary, 79.1% of marketed milk samples were contaminated, with an average AFM1 level of 18.0 ± 10.9 ng/kg, ranging from 5.3 to 100 ng/kg. AFM1 was detected in 9.4% of raw milk samples, and only one commercial sample (0.5%) exceeded the EU maximum residue limit (MRL) [26].

Referring to a previous study by Topi and colleagues (2022) [33], which presented data from 2019 to 2020, and comparing it with the findings of this study, we conclude that the incidence of AFM1 remains a constant concern in milk commodities produced in the country.

### 2.3. Exposure Assessment in Different Age Groups

**Figure 1:**
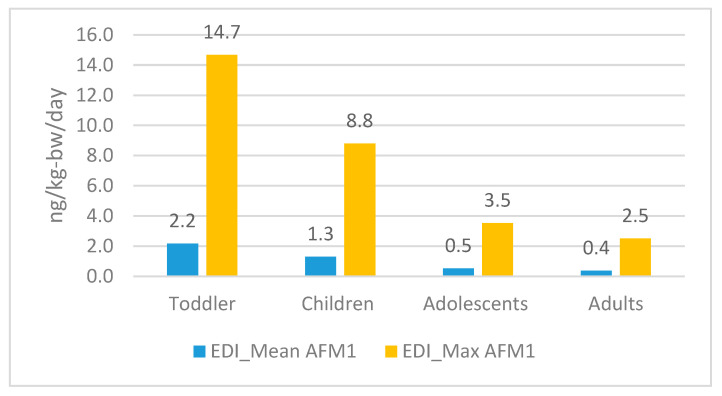
EDI values referring to exposure situation, on mean levels and maximum levels, for four different groups of populations.

Milk is an animal-derived food, making food contaminants unavoidable. Different regulatory bodies at the national and international levels monitor the presence of AFM1 in milk and dairy products. The concern over exposure to aflatoxin M1, a metabolite of AFB1, a Group 1 carcinogenic compound, has prompted health authorities to assess the risk and quantify the consequences of toxin exposure to humans of different ages. The average EDI, HI, MoE, and Risk Cancer values were calculated using the mean and maximum AFM1 levels for the entire study period (Table 2). Initially, the t-test for the two main groups, pasteurized and UHT milk samples, was conducted, concluding with *p* > 0.05, indicating that the difference was not considered significant. Based on this, the exposure assessment was conducted referring to the overall mean value of AFM1. Data is presented for four different population groups: toddlers (12 kg), children (20 kg), adolescents (50 kg), and adults (70 kg), with respective body weights according to EFSA (2011) [12]. The mean EDI values among adults were 0.370 ng/kg body weight/day; referring to the maximum AFM1 value, the EDI rose to 2.514 ng/kg body weight/day (Figure 1). Comparisons among the studied groups indicate a problematic situation for younger groups, with EDI based on mean AFM1 values being toddlers (2.161 ng/kg bw/day), children (1.297 ng/kg bw/day), adolescents (0.519 ng/kg bw/day), and adults (0.370 ng/kg bw/day). The newly calculated EDI is higher than that of a previous study, which found that adults’ EDI ranged from 0.082 to 0.096 ng/kg bw/day [33].

As an example, in Italy, the EDI value in Italian toddlers (0.16–0.52 ng/kg bw/day) and adults ranged from 0.02 to 0.08 ng/kg bw/day [18]; compared to this result, higher values were recorded in our study. The EDI values were also found to be higher than those for the adult population from Northern Macedonia (0.150 ng/kg bw/day) [29], Catalonia, Spain (0.305 ng/kg bw/day) [51], and Serbia (0.21 ng/kg bw/day) [30]. For Iranian male and female adults, the EDI values were 0.054 and 0.061 ng/kg bw/day, respectively, and significantly higher than the EDI of children, which is 0.119 ng/kg bw/day [52].

A correlation between consumers’ economic status and AFM1 exposure was documented by Ahlberg and colleagues (2018) [39] in their study on AFM1 among Kenyan consumers. They calculated the Estimated Daily Intake (EDI) for two groups with different socioeconomic statuses: low-income (1.2 ng/kg body weight/day) and mid-income (0.7 ng/kg body weight/day) consumers. The EDI among Indian consumers from the Ludhiana district, attributed to milk consumption, was 2.303 ng/kg body weight/day [44]. EDI values for Bulgarian toddlers and children ranged from 0.7 to 2.5 ng/kg bw/day and 0.5 to 1.9 ng/kg bw/day, respectively, raising concerns about the region’s health risk situation [12]. In another study assessing the average exposure of students to AFM1, EDI values ranged from 1.238 to 2.674 ng/kg bw/day for Serbia and 0.350 to 0.499 ng/kg−bw/day for Greece [53]. Considering students as adult consumers, we can conclude that the EDI value calculated in our study was lower compared to that of Serbian consumers and comparable to that of Greek consumers.

The Hazard Index (HI) and Margin of Exposure (MoE) are considered two of the most common indicators for assessing the risk of exposure to aflatoxins [52]. The HI values displayed an increasing trend among toddlers, children, adolescents, and adults, with mean values of 10.81, 6.48, 2.59, and 1.85, respectively, indicating AFM1 levels. The health risk assessment revealed that the study’s consumers, particularly children, are at a higher health risk from AFM1 due to their lower body weight and higher milk intake. Kuiper-Goodman (1990) [11] recommended the Hazard Index (HI) for the liver cancer risk associated with AFM1 exposure. HI higher than 1 ng/kg body weight indicates a considerable risk of liver cancer.

The Margin of Exposure (MoE) calculation indicates that only the adult group represents a value above 10,000, indicating a lower risk of aflatoxin M1 exposure, while the relationship between the risk of primary liver cancer and AFM1 exposure from milk and dairy product consumption has been demonstrated. The risk of hepatocellular carcinoma (HCC) cases per year per 10^5^ individuals across different age groups indicated that the HCC value using a deterministic approach was highest (0.234) and lowest (0.034) in the toddler age group (1–3 years). In the children age group (3–9 years), the values were 0.140 and 0.021, respectively; among adolescents aged (10–17 years), they were 0.55 and 0.008, respectively; and in the adult age group (>18 years), the values were 0.040 and 0.006, respectively. The HCC value was found to be higher compared to the EFSA reference on cancer risk, 0.004–0.007 [17]. Considering that consumer habits are very similar among different countries in Southeast Europe, we may consider that the health risk status is more severe compared with Northern Macedonian consumers (0.004) [29], Serbian consumers (0.0036–0.0047), and Greek consumers (0.0007–0.0009) [53].

The HCC values for different groups (Table 2), compared with the data from the study by Serraino and colleagues (2019) [18], indicate that cancer risk associated with milk consumption varies among toddlers (0.0032–0.0067), children (0.0011–0.0023), adolescents (0.0005–0.0010), and adults (0.0004–0.0008) per 100,000 individuals, respectively. These data show that exposure to AFM1 poses a higher risk, particularly for toddlers and children, due to their low body weight and high milk consumption rate, while the potential risk for hepatocellular carcinoma remains significant among Albanians.

Toddlers and children are at a higher biological risk due to higher milk intake per kilogram of body weight. Considering the focus of our study on the urban population, their high risk also stems from behavioral factors, as a specific characteristic of these groups is their daily presence in kindergartens and schools. Their diet consists of high amounts of milk and dairy products, indicating a greater exposure rate as well. Ongoing research by our team on food safety, particularly concerning the occurrence of mycotoxins in food and feed commodities within the country, suggests that governmental institutions need to play a key role in implementing good agricultural and manufacturing practices [21,22]. A national program for feed monitoring is necessary. To achieve this, raising awareness among policy-level actors requires attention.

## 3. Conclusions

This study presents the AFM1 levels and the exposure risk associated with milk consumption among different groups in Tirana, Albania. The analyzed milk indicates high exposure levels for Albanians, and addressing AFM1 contamination in dairy products is crucial for managing mycotoxin risk. The findings reveal a high rate of AFM1 contamination (62.4%) in the analyzed milk samples, with a notable proportion (26.2%) exceeding the European Union’s maximum residue levels. This raises concerns about food safety and public health, especially given the diverse population groups assessed, including vulnerable populations such as toddlers and children. The calculated Estimated Daily Intake (EDI) and Hazard Index (HI) values provide important insights into exposure levels and associated health risks, highlighting the urgent need for public health interventions. The MoE for four age groups demonstrates a significant health risk, with toddlers and children exhibiting the highest vulnerability. Considering these results, stricter surveillance programs and legislation must be implemented to reduce the population’s exposure to these risks.

## 4. Materials and Methods

### 4.1. Sample Collection

A total of 141 cow milk samples were purchased from the local markets of Tirana between March 2023 and February 2024. The samples consisted of raw milk (*n* = 13), pasteurized milk (*n* = 64), and UHT milk (*n* = 64). The sampling strategy reflected the tradition of local farmers marketing raw milk to urban consumers, while pasteurized and UHT samples, both categories equally represented in the study, aimed to avoid statistical production data but to estimate the risk exposure to consumers. Finally, the collected samples were stored in a refrigerator at 4 °C prior to analysis for the presence of AFM1.

### 4.2. Chemicals and Standards

The analytical standard of AFM1 was supplied by RomerLab (Vienna, Austria). All solvents used to prepare the mobile phase were HPLC grade and obtained from Fluka (Brussels, Belgium). All homogenized mixtures and eluates were filtered through Whatman no. 4 and 0.45 mm (Whatman plc, Maidstone, UK). Deionized water was obtained with a Millipore Elix Essential purification system (Bedford, MA USA). AflaCean AFM1 immunoaffinity columns (stored at 4 °C until use) were supplied by LGC (Wesel, Germany) and used for sample cleanup.

### 4.3. Preparation of Standard Solutions

A mother stock solution (# 10000349 Aflatoxin M1—0.5 µg/mL, 5 mL) was purchased from RomerLabs and stored in a freezer (−20 °C). A working stock solution of 0.01 μg/mL was diluted stepwise with the mobile phase (acetonitrile/water, 75/25, *v*/*v*) to prepare a sequence of working solutions, which were stored in vials at a temperature below 4 °C for calibration curve preparation. Calibration solutions of 0.02 μg/kg, 0.04 μg/kg, 0.06 μg/kg, 0.08 μg/kg, and 0.10 μg/kg were used. Samples with AFM1 amounts exceeding the calibration range were diluted, and dilution factors were applied for quantification, as outlined in EN ISO 14501:2021 [54].

### 4.4. Preparation of Samples

After warming at about 37 °C in water, both samples were centrifuged at 2000 g/min to separate the fat layers and then filtered. The prepared test portion of 50 mL was transferred into a syringe barrel attached to the AFM1 immunoaffinity column (IAC) and passed at a slow, steady flow rate of 1–2 mL/min. The columns were washed with 20 mL of deionized water, and the air was passed through them to dryness.

AFM1 was eluted with acetonitrile/MeOH (60:40 *v*/*v*) by allowing it to contact the column for at least 60 s. The eluate was evaporated to dryness using a nitrogen stream. The residue was dissolved in 1 mL of mobile phase and filtered using a membrane filter before being injected into HPLC for quantification.

### 4.5. HPLC-FLD Method for AFM1 Analysis

An Agilent high-performance liquid chromatography system (HPLC 1260 Infinity series) with a quaternary pump and fluorescence detection was used for AFM1 quantification analysis. Data acquisition and quantification were performed using ChemStation (OpenLab edition). The analytical method was adapted according to the standard method EN ISO 14501:2021 [54]. The Agilent HPLC equipped with a fluorescence detector was set at an excitation wavelength of 360 nm and an emission wavelength of 440 nm. The column compartment (HPLC Column: TC-C18 (2), 170, 5 μm, 4.6 × 250 mm; thus, pore size of 170, particle size of 5.0 μm, inner diameter of 4.6 mm, length of 250 mm, and carbon load of 12%) and column temperature were regulated at 30 °C. The mobile phase was a mixture of water and acetonitrile (60:40, *v*/*v*), and an isocratic delivery mode was employed at a flow rate of 1 mL/min and an injection volume of 100 µL.

### 4.6. Validation

The HPLC-FLD method was validated in accordance with the guidelines established by the European Commission Implementing Regulation (EU) 2023/2782 [55] for the analysis of mycotoxin levels in food. The method was evaluated for linearity, the limit of detection (LOD), the limit of quantification (LOQ), accuracy, precision, and selectivity. The accuracy of the method was assessed by preparing five-point solvent-matched calibrations in triplicate for AFM1 standard solutions within the concentration range of 0.01–4.0 µg/kg. Calibration curves were generated by plotting the peak area against the AFM1 concentration, and the linearity was determined using linear regression analysis, expressed as the coefficient of determination (R^2^ = 0.998).

The method’s precision was determined by calculating the percentage relative standard deviation (% RSD) of three identical extractions of milk samples spiked with AFM1 at the same and three different spiking levels. The method’s selectivity was evaluated by analyzing AFM1 in a known negative milk matrix and reagent blank to ensure the absence of any interference from endogenous substances around the retention time of the target analyte (Table 3).

### 4.7. Risk Assessment

#### 4.7.1. Estimated Daily Intake

The risk assessment of AFM1 through milk consumption was estimated daily and referred to as Estimated Daily Intake (EDI).

$$EDI(\frac{ng}{kg}-bw/day)=\frac{C_{AFM1}(ng/L)\times DMI(L/day)}{Bw(kg)}$$

C_AFM1_ refers to the AFM1 concentration in milk samples (ng/kg). DMI is the average daily milk consumption (DMI), which was 441.05 mL/day per Albanian consumer, according to the WHO/GEMS database, Cluster G02 [56]. BW is the consumer’s body weight (Table 4), as defined by EFSA [12].

#### 4.7.2. The Hazard Index (HI)

The Hazard Index (HI) value indicates the emerging health risk from AFM1 exposure through milk, calculated using the Estimated Daily Intake (EDI) and Reference Dose (RfD).

$$HI=\frac{EDI(ng/kg-bw/day)}{RfD(ng/kg-bw/day)}$$

The safety dose, 0.2 ng/kg body weight/day, was described as the HI derived from AFM1-contaminated milk consumption. HI levels over 1 indicate a significant health risk of exposure to AFM1 in the milk-consuming population [11].

### 4.8. Risk Characterization

#### 4.8.1. Margin of Exposure

The risk characterization derived from milk consumption in different age groups of Albanians was estimated through the Margin of Exposure (MoE) according to the EFSA. The MoE represents a risk index derived from oral exposure to compounds with carcinogenic and genotoxic activity [17]. The MoE value for each age group was calculated using the equation below.

$$MoE=\frac{{BMDL}_{10}}{EDI(ng//kg-bw/day)}$$

The BMDL_10_ represents the benchmark dose of the toxic substance responsible for increasing the incidence of liver cancer by 10%. A value of MoE < 10,000 reflects a worrying risk level of liver cancer in a specific group induced by AFM1 exposure. The EFSA has approved the application of a potency factor of 0.1, combined with the BMDL_10_ (0.4 µg/kg bw per day), for the induction of liver cancer by AFB1. This approval concludes the application of the value (4 µg/kg bw/day) in the present study for the AFM1 risk assessment.

#### 4.8.2. Liver Cancer Risk Characterization

The fraction of hepatocellular carcinoma (HCC) was calculated according to Serraino et al. (2019) [18], assuming that hepatitis B (HBV) carriers among Albanians potentially vary by 2%. According to JECFA, the cancer potencies for aflatoxin B1 are 0.01 for individuals who are hepatitis B surface antigen-negative (HBsAg¯) and 0.3 for those who are hepatitis B surface antigen-positive (HBsAg^+^) [57].

$$Pcancer=(0.01\times {HBsAg}^{-})+(0.3\times {HBsAg}^{+})$$

According to the given cancer potency, the risk of cancer is calculated as follows:

Pcancer=(0.01×0.98)+(0.3×0.02)=0.0158

The cancer risk among Albanians was calculated to be 0.0158 HCC per 100,000 persons per year. The proportion of the population at risk was estimated by multiplying the risk potency by the BMDL_10_ and then dividing by the MoE, considering the mean of exposure estimation [57].

$$Populationatrisk=\frac{riskpotency\times {BMDL}_{10}}{MoE}$$

## Figures and Tables

**Table 1:** Aflatoxin M1 incidence in cow milk in Albania.

Parameter	Incidence (All Samples)	Percentage (%)
Positive samples	88 (141)	62.4
Interval < LOD (3 ng/kg)	53 (141)	37.6
Interval 3–10 ng/kg	21 (141)	14.9
Interval 10–50 ng/kg	30 (141)	21.3
Above EU MRL (>0.05 µg/kg)	37 (141)	26.2
Min-Max range (ng/kg)	3.0–399.0	
Mean ± StDev (ng/kg)	58.80 ± 95.87	
Median (ng/kg)	11.0	

**Table 2:** Risk assessment of AFM1 exposure through milk consumption in Tirana, Albania.

Value	Toddler (12 kg)	Children (20 kg)	Adolescents (50 kg)	Adults (70 kg)
AFM1 (ng/kg)	Mean (Max)	Mean (Max)	Mean (Max)	Mean (Max)
EDI (ng/kg bw/day)	2.161 (14.666)	1.297 (8.800)	0.519 (3.520)	0.370 (2.514)
HI (ng/kg bw/day)	10.81 (73.33)	6.48 (44.00)	2.59 (17.60)	1.85 (12.57)
MoE	1850.9 (272.7)	3084.0 (454.5)	7707 (1136.4)	10,810.8 (1590.9)
Population at risk of HCC	0.034 (0.234)	0.021 (0.140)	0.008 (0.055)	0.006 (0.040)

Mean value; Maximum value; HI—Hazard Index; MoE—Margin of Exposure; HCC—hepatocellular carcinoma.

**Table 3:** Recovery and precision data of the applied HPLC-FLD method of AFM1 analysis in milk.

LOD (μg kg^−1^)	LOQ (μg kg^−1^)	Spiked Level (μg kg^−1^)	Repeatability (*n* = 3)			
			Day 1		Day 2	
			Mean Recovery (%)	RSD (%) *n* = 3 (Milk)	Mean Recovery (%)	RSD (%) *n* = 3 (Milk)
0.003	0.010	0.01	101.5	5.93	95.2	4.78
		0.05	92.4	4.8	93.1	4.27
		0.1	84.1	3.92	86.2	4.52

**Table 4:** European country population group description [12].

Age Group	Age	Body Weight
Toddlers	12–35 months	12
Other children	3–9 years	20
Adolescents	10–17 years	50
Adults	18–64 years	70

## Data Availability

The original contributions presented in this study are included in this article. Further inquiries can be directed to the corresponding author.
